# ^18^F-fluoride positron emission tomography/computed tomography and bone scintigraphy for diagnosis of bone metastases in newly diagnosed, high-risk prostate cancer patients: study protocol for a multicentre, diagnostic test accuracy study

**DOI:** 10.1186/s12885-016-2047-1

**Published:** 2016-01-11

**Authors:** Randi F. Fonager, Helle D. Zacho, Niels C. Langkilde, Lars J. Petersen

**Affiliations:** Department of Nuclear Medicine, Clinical Cancer Research Center, Aalborg University Hospital, Hobrovej 18-22, DK-9000 Aalborg, Denmark; Department of Urology, Aalborg University Hospital, Reberbansgade 15, DK-9000 Aalborg, Denmark; Department of Clinical Medicine, Aalborg University, Sdr. Skovvej 11, DK-9000 Aalborg, Denmark

**Keywords:** Positron emission tomography/computed tomography, ^18^F-fluoride, Planar bone scintigraphy, Bone metastases, Prostate cancer

## Abstract

**Background:**

For decades, planar bone scintigraphy has been the standard practice for detection of bone metastases in prostate cancer and has been endorsed by recent oncology/urology guidelines. It is a sensitive method with modest specificity. ^18^F-fluoride positron emission tomography/computed tomography has shown improved sensitivity and specificity over bone scintigraphy, but because of methodological issues such as retrospective design and verification bias, the existing level of evidence with ^18^F-fluoride positron emission tomography/computed tomography is limited. The primary objective is to compare the diagnostic properties of ^18^F-fluoride positron emission tomography/computed tomography versus bone scintigraphy on an individual patient basis.

**Methods/Design:**

One hundred forty consecutive, high-risk prostate cancer patients will be recruited from several hospitals in Denmark. Sample size was calculated using Hayen’s method for diagnostic comparative studies. This study will be conducted in accordance with recommendations of standards for reporting diagnostic accuracy studies. Eligibility criteria comprise the following: 1) biopsy-proven prostate cancer, 2) PSA ≥50 ng/ml (equals a prevalence of bone metastasis of ≈ 50 % in the study population on bone scintigraphy), 3) patients must be eligible for androgen deprivation therapy, 4) no current or prior cancer (within the past 5 years), 5) ability to comply with imaging procedures, and 6) patients must not receive any investigational drugs. Planar bone scintigraphy and ^18^F-fluoride positron emission tomography/computed tomography will be performed within a window of 14 days at baseline. All scans will be repeated after 26 weeks of androgen deprivation therapy, and response of individual lesions will be used for diagnostic classification of the lesions on baseline imaging among responding patients. A response is defined as PSA normalisation or ≥80 % reduction compared with baseline levels, testosterone below castration levels, no skeletal related events, and no clinical signs of progression. Images are read by blinded nuclear medicine physicians. The protocol is currently recruiting.

**Discussion:**

To the best of our knowledge, this is one of the largest prospective studies comparing ^18^F-fluoride positron emission tomography/computed tomography and bone scintigraphy. It is conducted in full accordance with recommendations for diagnostic accuracy trials. It is intended to provide valid documentation for the use of ^18^F-fluoride positron emission tomography/computed tomography for examination of bone metastasis in the staging of prostate cancer.

## Background

Prostate cancer is one of the most frequent cancers in men [1]. It often metastasises to the bone, and this is associated with significant morbidity and mortality [2, 3]. According to current urology and oncology guidelines, planar bone scintigraphy (BS) remains the standard practice for detection of bone metastases in prostate cancer [4–6]. BS has a high sensitivity for detection of bone metastases in the staging of prostate cancer while its specificity is moderate. Activity on BS may also represent benign conditions such as degenerative bone disorders, traumas and inflammatory conditions [7]; these conditions frequently occur in older men diagnosed with prostate cancer [8].

Technical development within nuclear medicine bone imaging has emerged since the introduction of BS, including single photon emission computed tomography/computed tomography (SPECT/CT), acquisition of BS, and positron emission tomography/computed tomography (PET/CT) with ^18^F-fluoride and ^18^F- or ^11^C-choline [9, 10]. The principle of ^18^F-fluoride is somewhat similar to BS since it reflects regenerative bone processes, not the bone metastasis itself. However, compared with the BS tracer, ^18^F-fluoride has a higher bone uptake, a faster blood clearance and an improved target-to-background ratio [11]. Furthermore, PET is associated with higher spatial resolution than gamma-camera-based BS and likely may improve diagnostic accuracy [11–13].

Retrospective studies, often with a limited number of patients, have indicated that ^18^F-fluoride PET/CT is superior to BS for detection of bone metastases in patients with newly diagnosed prostate cancer and patients with recurring prostate cancer. However, in the absence of histopathological verification, the definitions of presence or absence of bone metastases are essential for interpretation of diagnostic comparative studies. A recent systematic review identified this issue as a key methodological flaw in studies with bone-targeting PET ligands [10]. Thus, the advantage of ^18^F-fluoride PET versus BS for the diagnosis of bone metastases remains to be shown in well-designed studies.

The primary aim of this diagnostic test accuracy study is to compare ^18^F-fluoride PET/CT versus guideline-recommended BS in diagnosing bone metastases in newly diagnosed prostate cancer. The study protocol is in full compliance with recommendations for diagnostic test accuracy studies [14, 15]. Particular attention is aimed at applying an optimised reference standard, i.e., confirming the presence or absence of bone metastases.

## Methods/Design

### Study objectives

The primary objective of this study is to assess the diagnostic accuracy of ^18^F-fluoride PET/CT for detection of bone metastases compared with BS in newly diagnosed, high risk, untreated prostate cancer patients on an individual patient basis.

Secondary objectives are: 1) to assess the diagnostic properties of SPECT/CT in comparison with BS and ^18^F-fluoride PET/CT, 2) to evaluate the diagnostic properties of all imaging modalities on the basis of individual lesions, 3) to investigate the inter- and intra-observer variation of SPECT/CT and ^18^F-fluoride PET/CT, and 4) to investigate the predictive role of bone tumour load as measured by ^18^F-fluoride PET/CT as a predictor of time to loss of hormone sensitivity.

### Study design

This study is designed as a multicentre, single-group, prospective diagnostic test accuracy (DTA) study. It will be conducted according to methodological criteria and recommendations as outlined by Standards for Reporting of Diagnostic Accuracy studies (STARD) [14] and the Grading of Recommendation, Assessments, Development and Evaluation (GRADE) [15].

Within a time window of 14 days, and no later than 7 days after initiation of androgen deprivation (ADT), consenting patients will be examined by BS, SPECT/CT and ^18^F-fluoride PET/CT. These scans are baseline examinations (Table 1). To assist in determination of equivocal lesions on the baseline scan, the response to treatment will be examined after 6 months of ADT. Studies indicate regression of bone metastasis within 6 months following ADT in prostate cancer [16–21], notable decrease of baseline PSA within 6 weeks [16] and testosterone levels below castration levels (≤50 ng/mL) within 4 weeks. The time period of 6 months should be sufficient to demonstrate notable treatment effects without any influence of the short-lasting, treatment-induced osteoblastic response called the flare phenomenon [22, 23].

**Table 1:** Overview of study procedures

	Baseline		Follow-up	
Visit number:	1	1a	2	2a
Day:	0	1	180	181
Androgen deprivation theapy	Ongoing			
Planar bone scintigraphy	X		X	
SPECT/CT	X		X	
^18^F-fluoride PET/CT		X		X
PSA	X		X	
P-testosterone	X		X	

**Fig. 1:**
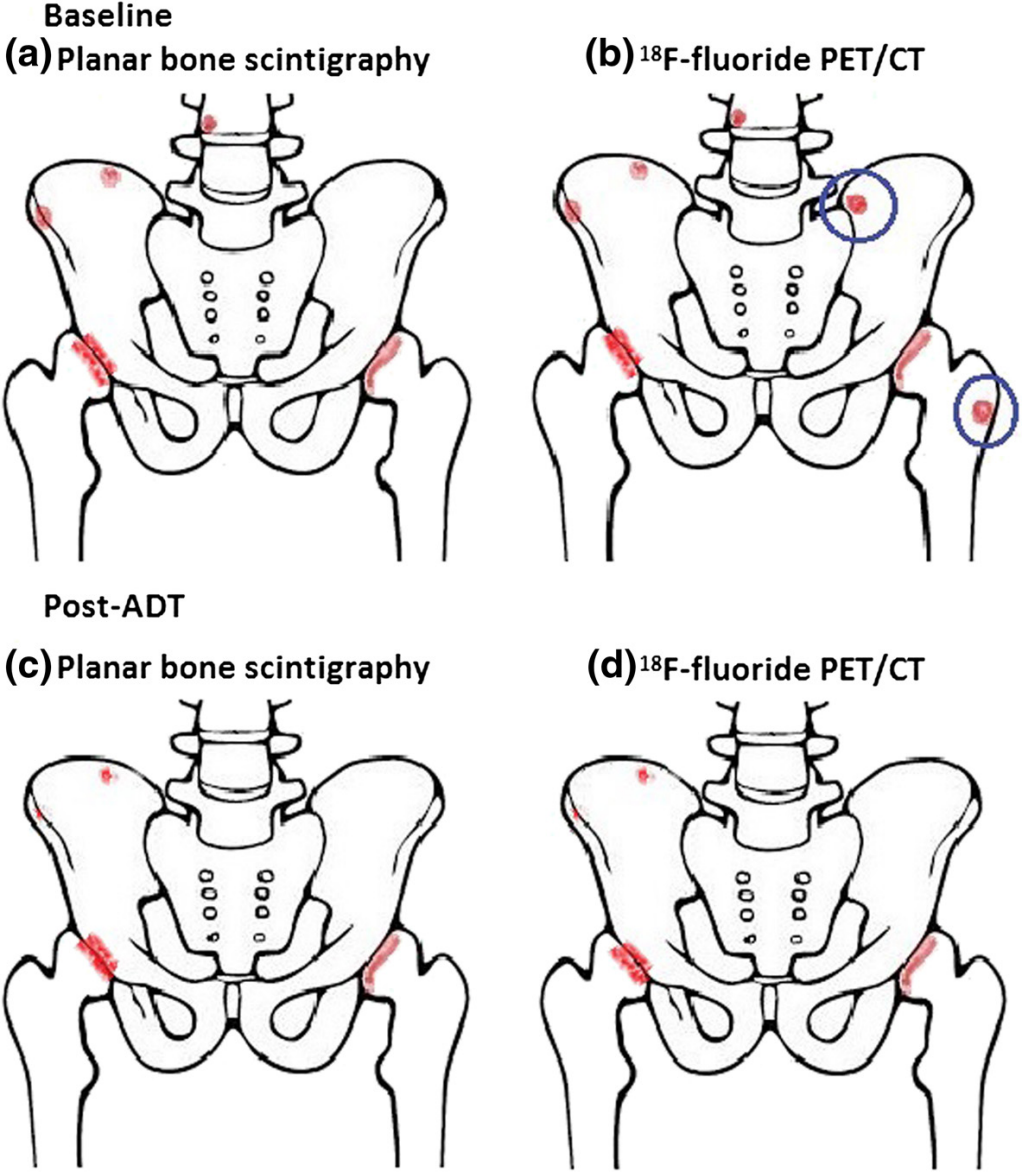
Schematic drawing of the treatment response of planar bone scintigraphy and ^18^F-fluoride PET/CT. **a** Baseline imaging with planar bone scintigraphy (BS); BS shows two lesions in the pelvic region, one lesion in a vertebra, and lesions at both hip joint surfaces. **b** ^18^F-fluoride PET/CT demonstrate two additional lesions that were not detected by BS (marked with blue circle). **c** Post-ADT imaging with BS. **d** Post-ADT imaging with ^18^F-fluoride PET/CT. All lesions detected by BS, which are not located near joints, showed partial (*n* = 2) or complete (*n* = 1) regression and thus were defined as bone metastases. All the lesions detected by ^18^F-fluoride PET/CT, which are not located near joints, regressed (*n* = 5). Thus, ^18^F-fluoride PET/CT detected two lesions that were not detected by BS, these are defined as true positive on^18^F-fluoride PET/CT and consequently as false negative on BS. *ADT* androgen deprivation therapy. The illustration is copyright of Nuclear Medicine Aalborg

**Fig. 2:**
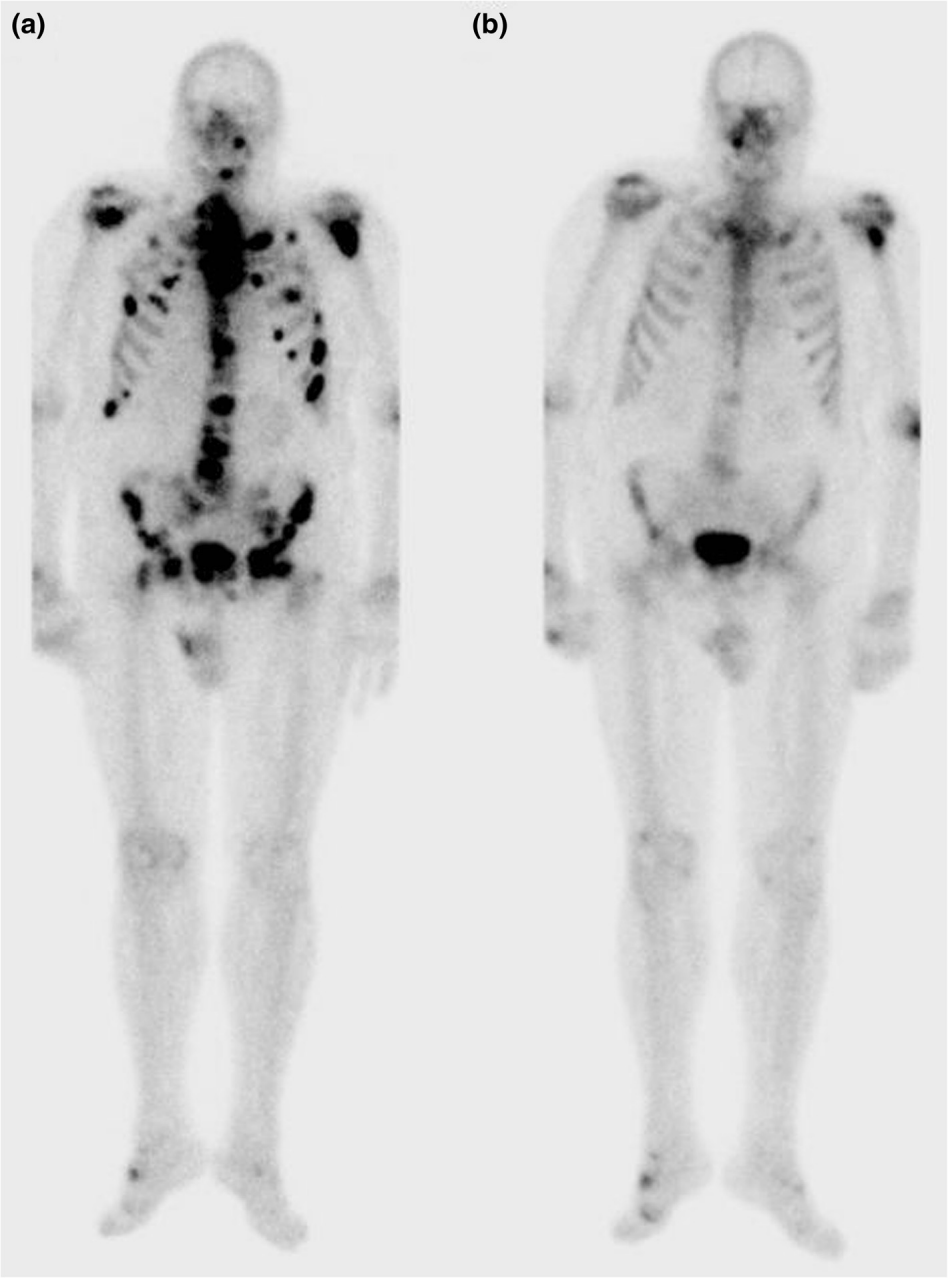
Pre- and post-androgen deprivation therapy images from planar bone scintigraphy (anterior view). Anterior images from planar bone scintigraphy of one patient at baseline (**a**) and after 6 months of satisfactory androgen therapy (**b**). PSA decreased from 92 ng/mL at baseline to 8.8 ng/mL (90 % reduction), and plasma-testosterone decreased from 1.7 to 0.07 ng/ml. All lesions initially suspected of malignancy in the axial skeleton demonstrated partial or complete regression, whereas lesions in large joints and small joints in the hands and feet were stable or progressed. The activity in the left elbow region is an artifact caused by contamination at tracer injection

Following 6 months of ADT, patients with a satisfactory response will have all three scans repeated (Table 1). A satisfactory ADT response is defined as: 1) normalisation of PSA or at least 80 % reduction of baseline PSA levels, 2) plasma-testosterone below castration levels, 3) no skeletal-related events since baseline, and 4) no clinical, biochemical, or other indication of disease progression. The imaging response to satisfactory ADT will guide the readers to classify metastasis and benign lesions on the baseline scans (Fig. 1). ADT affects both the primary tumour and bone metastatic cancer cells, and bone metastases will therefore regress or become indistinguishable on imaging following satisfactory ADT [29, 30] (Fig. 2). The patients will be followed clinically until the cancer has progressed to castration-resistant prostate cancer according to criteria from the European Association of Urology [26].

### Study population

A total of 140 consecutive patients will be recruited. Eligibility criteria comprise: 1) biopsy-proven prostate cancer, 2) PSA ≥50 ng/ml, 3) patients must be eligible for androgen deprivation therapy, 4) no current or prior cancer (within the past 5 years), 5) ability to comply with imaging procedures, and 6) no investigational drugs. Based on existing data, the prevalence of bone metastases in this population is expected to be approximately 50 % on BS [8]. A bone metastasis-enriched population was selected to optimise sample size while still taking into consideration obtaining a reference test. Inclusion of patients scheduled for prostatectomy or radiation therapy would interfere with the definitions of the reference standard and presence or absence of bone metastasis (see Rationale for design).

Subjects will be recruited consecutively from, at present, four urological departments in Denmark.

### Ethical considerations

This DTA study will be conducted according to the principles of the Helsinki II Declaration. The patients receive oral and written information about the study and provide written informed consent prior to any study-related procedures. The study protocol is approved by the North Denmark Region Committee on Health Research Ethics (N-20130068) and the Danish Data Protection Agency.

### Imaging procedures

#### Planar bone scintigraphy and SPECT/CT

BS and SPECT/CT is conducted in accordance with current institutional recommendations which are in line with international guidelines [27]. Whole body BS is performed 2 h after injection of approximately 750 M Becquerel (MBq) 99^m^Tc-labelled diphosphonate. Three-bed SPECT/CT torso-scan (from vertex to mid-thigh) is performed immediately after BS. The CT component used with SPECT as well as with PET is a low-dose acquisition primarily used for attenuation correction and anatomical localisation.

### ^18^F-fluoride PET/CT

^18^F-fluoride PET/CT will be conducted in accordance with recent American and German guidelines [28, 29]. PET/CT will be performed approximately 30 min after intravenous administration of 200 MBq ^18^F-fluoride. A total of 7 to 9 bed positions are performed with an acquisition time of 2.5 min per bed position resulting in a scan from the skull to mid-thigh.

### Image analysis

Diagnostic accuracy is primarily analysed on a patient-basis. All images are evaluated by a reading committee of two readers who must be board certified in Nuclear Medicine and/or Radiology and experienced with the imaging modality. Readers will have access to PSA, T-stage, Gleason grade, and a standard questionnaire filled out by the patients. The amount of clinical information will be minimised to reduce reading bias but the amount of information will be sufficient to reflect clinical practice and thus, will present the generalisability of the findings. The questionnaire contains information about any artificial joint replacements, prior surgeries to joint or bone, prior skeleton or joint infections, known degenerative or inflammatory bone diseases, recent trauma to the skeleton, and location and duration of any bone pain [8].

All lesions, or a representative sample if a large number of lesions are present, are first classified independently by each reader for malignancy using a dichotomous scale as well as a numerical rating scale which includes an equivocal rating option [30]. The latter scale will be used primarily to determine observer agreement. Eventually, the readers will reach consensus for the dichotomous outcome. If consensus cannot be reached, a third reader will be included and a majority rule will apply. Lesion analysis is then summarised on an individual patient basis. The exact measurement scales and how to handle patients with multiple bone lesions will be stated in a reader manual and the statistical analytical plan (see Data Analysis). Readers may participate in the reading committee for more than one imaging modality.

### Image evaluation procedure

#### Baseline images

A standardised protocol for image analysis will be used. As a rule of thumb, the following applies: 1) lesions located in the pelvis or spine, which are not directly joint-related (e.g., sacroiliac joint, facet joints, or discs) are malignant, 2) isolated lesions outside the pelvic/hip area, with no simultaneous uptake in the axial skeleton, are benign; however, if concurrent metastases in the axial skeleton are present, malignancy is considered, 3) lesions in hands, feet, and at large joints (shoulders, elbows, hips joints, and knees) are benign.

Even though the use of low-dose CT is primarily for attenuation correction and anatomical localisation, any diagnostic information obtained from the CT scan will be used, e.g., the characteristics and extent of osteosclerotic and osteolytic lesions, lesion irregularity, etc.

#### Follow up images

Once the readers have assessed the baseline scan for a patient (and the case report form has been signed), they read baseline and 6 month images for that patient side-by-side. Based on a subjective evaluation of lesion characteristics at baseline along with lesion changes from baseline to 6 months, the readers will classify individual lesions as malignant or benign. In equivocal or inconsistent cases, baseline and follow-up images from all three imaging modalities will be read and evaluated together. Any new lesions seen on the follow-up scan but not observed on the baseline image on any imaging modalities will not be classified.

#### Final diagnosis

A map of lesions identified by all imaging modalities will be drawn. Some lesions may be observed on all imaging modalities, while other lesions may be observed only in one or two modalities. The lesions will be analysed per modality and combined and classified as: 1) **True positive:** A lesion that was defined as M+ on the baseline image and that responded to ADT on on any follow-up images, 2) **False positive:** A lesion that was defined as M+ on the baseline image but did not respond to ADT in any of the imaging modalities, 3) **False negative:** A lesion that was not identified on one imaging modality at baseline, but turned out to eventually be classified as M+ on other imaging modalities, e.g. a lesion that was not detected by BS but detected and classified as true positive on ^18^F-fluoride PET/CT will be classified as false negative on BS, see Fig. 1, and 4) **True negative:** A lesion that was not identified on one imaging modality at baseline, and was eventually classified as M- on other imaging modalities as well. Patients with at least one lesion characterised as malignant by any imaging modality will be classified as malignant on an individual patient basis. How to handle inconsistent responses between imaging modalities will be specified in the statistical analysis plan. For lesions-based analysis, final diagnosis is determined by the same criteria as for the patient-based analysis, as described above.

### Sample size considerations

Sample size calculations are based on recommendations from Hayen et al. for DTA studies [31]. Weighed means of sensitivity and specificity of BS, SPECT/CT and ^18^F-fluoride PET/CT were calculated based on reported values in published clinical trials. The power calculation showed that 114 patients are needed to identify a significant difference between the false positive fractions (i.e., 1-specificity) of BS and SPECT/CT versus ^18^F-fluoride PET/CT with a type I error of 5 % and a type II error of 20 %, assuming a prevalence of bone metastases of 50 % on BS. A total of 140 patients will be recruited to account for possible dropouts. Calculated weighed mean values of true positive fractions (sensitivity) for BS, SPECT/CT and ^18^F-fluoride PET/CT are very similar (0.87, 0.90, and 0.87, respectively), which indicated that more than 5,000 patients were needed to demonstrate a significant difference in sensitivity among the methods.

### Data analysis

A detailed statistical analysis plan, including considerations for secondary endpoints, will be issued prior to analysis. Data analysis will primarily focus on the diagnostic accuracy of BS and ^18^F-fluoride PET/CT. Sensitivity, specificity, positive and negative predictive values, and likelihood ratios will be calculated for each imaging modality with 95 % confidence intervals and will be compared using the McNemar test, with *P* < 0.05 being statistically significant.

### Quality assessment

All scans are performed according to local practices which are in line with international guidelines [29]. No detailed requirement for accreditation of the equipment prior to baseline is applied. However, the image quality of the applied scanners is compared in order to adjust for any relevant differences in scanner performances in the statistical analysis. The following performance measurements will be obtained: data from the initial installation of the scanner, data from the most recent quality control, and prospective, study-related data from phantom scans.

### Rationale for design

#### Patient selection

The GRADE recommendations state that valid DTA studies should include representative and consecutive patients [15]. This study is conducted in bone-metastasis-enriched patients with prostate cancer due to the ADT-assisted definition of the presence or absence of bone metastasis. However, inclusion of low-risk patients as well as patients undergoing curatively intended treatment would interfere with sample size calculations and/or methodological issues with regard to the validity of the reference standard. For example, persistently elevated PSA levels following radical prostatectomy may arise from the remnant primary tumour or lymph nodes, as well as bone metastases. In addition, the PSA response in patients receiving curatively intended radiation therapy may be very slow, may be masked by concomitant ADT; any progression some years after post-therapy cannot be attributed with certainty to bone metastases at the time of diagnosis.

### DTA design

This DTA protocol and the planned manuscript is and will be in full compliance with the 25 items of the STARD guideline, including title and abstract, introduction, methods (participants, test methods, statistical analysis), and results. Similar to the CONSORT statement of reporting of randomised controlled trials and the PRISMA statement for reporting of systematic reviews (see www.equator-network.org), STARD is a guideline for reporting of DTA studies. The trial methodology laid down in the STARD recommendations have also been endorsed by the Cochrane organisation for systematic reviews of diagnostic test studies. It is generally accepted that properly conducted DTA trials are a requirement before the conduct of randomised controlled trials to study the impact of different diagnostic strategies on patient outcome [15, 32]. This DTA study is completely compliant with STARD criteria [14]. Compliance with and reporting of STARD items in DTA studies has been slowly increasing since the introduction of STARD; however, according to Korevaar et al., as of 2014, reporting of STARD could still be improved [33].

According to GRADE, patient-important outcomes can be inferred on the basis of diagnostic test accuracy. This means that if this DTA study demonstrates that the diagnostic accuracy is significantly improved by ^18^F-fluoride PET/CT compared with BS then more cases of prostate cancer will be correctly classified according to disease stage, thus ensuring optimal management of the disease. On the contrary, if the diagnostic accuracy is equal for both modalities but ^18^F-fluoride PET/CT is more convenient for the patient (e.g., shorter time from injection to scan and shorter scan time), these results will ensure that the course of diagnosis is optimal.

## Discussion

Early and correct diagnosis of bone metastases in prostate cancer is important for clinical decision making. Thus, sensitive and specific diagnostic techniques are required. BS remains the guideline-recommended method for staging of bone metastasis in prostate cancer, but it can be debated if this is appropriate in light of emerging, interesting methods such as ^18^F-fluoride PET/CT and multi-parametric magnetic resonance imaging [34].

The decision to select an appropriate diagnostic method preferably should be made based on evidence-based recommendations. However, in 2011, Poonacha et al. [35] published a study examining the level of evidence underlying clinical recommendations from the National Comprehensive Cancer Network. It was revealed that no recommendations for prostate cancer staging, as well as for any other diagnostic recommendations across tumour types, were based on level I evidence. Small series and retrospective studies have indicated that ^18^F-fluoride PET/CT is significantly better than BS; however, the superiority of ^18^F-fluoride PET/CT remains to be shown in properly designed and well-powered clinical trials [9, 13, 36–38]. The level of evidence among previously published studies is quite low (level 3b according to the Oxford Centre for Evidence-based Medicine) [10]. The low level of evidence in diagnostic medicine is a general phenomenon [35], but the issues have been highlighted on several occasions regarding imaging [33]. We believe that a large DTA study performed in accordance with STARD recommendations will allow us to make firm conclusions about the diagnostic properties and potential advantages of ^18^F-fluoride PET/CT versus guideline-recommended BS.

We realise that strict methodological criteria and high quality procedures may conflict with generalisability of the findings in clinical practice. Therefore, images are read with key clinical information as would be available in clinical situations. The risk of reading bias is present but the reading conditions are fully described; thus, all stakeholders can judge the results based on his or her premises. Similarly, gamma cameras and PET/CT scanners are not accredited or standardised prior to recruitment as required in some multicentre trials, e.g., those from the European Organization on Research and Treatment of Cancer. Instead, we aimed at comparing the imaging modalities as they are used in daily clinical practice rather than comparing the imaging modality per se under optimal instrumental settings.

^18^F-fluoride PET/CT has already been routinely applied for detection of bone metastases in prostate cancer in some clinics. However, it is important to note that in the most recent guidelines from the National Comprehensive Cancer Network on prostate cancer, panelists express their concern about the inappropriate use of expensive PET imaging in the clinical setting [39], e.g., ^18^F-fluoride PET/CT for the staging of prostate cancer. Thus, there is a rationale for conducting properly designed DTA studies before making changes in clinical practice. If superiority is clearly evident, the work required to demonstrate it is limited. The design and size of this study ensure that the results will be recognised both nationally and internationally; the perspective may be the general use of ^18^F-fluoride PET/CT for bone imaging in prostate cancer.

## Trial status

Recruiting.

## Abbreviation

ADT: androgen deprivation therapy
BS: planar bone scintigraphy
DTA: diagnostic test accuracy
GRADE: the grading of recommendations assessment, development and evaluation
PET/CT: positron emission tomography/computed tomography
PSA: prostate specific antigen
RCT: randomised controlled trial
SPECT/CT: single photon emission computed tomography/computed tomography
STARD: standard for the reporting of diagnostic accuracy studies
